# Supplementary material for: Geometric graphs from data to aid classification tasks with Graph Convolutional Networks
Source: Patterns (N Y). 2021 Apr 9;2(4):100237. doi: 10.1016/j.patter.2021.100237 (PMC8085612; doi:10.1016/j.patter.2021.100237)
Supplement: Document S1. Figures S1–S6, Tables S1–S6, and Notes S1–S5 [file mmc1.pdf]

Patterns, Volume *2*

Supplemental information

Geometric graphs from data to aid classification tasks with Graph Convolutional Networks

Yifan Qian, Paul Expert, Pietro Panzarasa, and Mauricio Barahona

Supplemental Notes

Note S1: Data sets

We use seven data sets collected from various sources. We provide the data sets at https://github.com/haczqyf/ggc/tree/master/ggc/data. The data set statistics are summarized in Table S1.

1. *Constructive*[1] is a synthetic data set generated by a stochastic block model that reproduces the ground truth structure with some noise. Each ground truth cluster is associated with 50 features with a probability of $p_{\text{in}} = 0.07$ equal to 1. Each sample also has a probability of $p_{\text{out}} = 0.007$ of possessing each feature characterizing other clusters.

2. We consider two data sets with text documents: *Cora*[1,2] and *AMiner*[3,4]. In Cora and AMiner, the samples are scientific papers where each paper is associated with a high-dimensional bag-of-words feature vector extracted from the paper content. Each sample has a class label indicating its scientific field.

3. *Digits* is a handwritten digits data set. Each sample is a 8x8 image of a digit. This is one of the benchmark data sets for classification in Scikit-learn[5].

4. *FMA*: The original data set[6,7] contains 140 audio features extracted from $7,994$ music tracks. We use this data set to address the problem of genre classification. The original data set in Ref.[6] contains 8 genres. We sample randomly $2,000$ music tracks (250 for each genre) to produce our data set.

5. *Cell*: This is a data set of brain cell types from autism. The original data set[8] contains the gene expression values ($\log_2$ transformed 10x UMI counts from cellranger) of $104,599$ single cells from brains of control individuals and of patients with autism, where each cell (sample) is characterized by the expression level of $36,501$ genes (features). The full data set contains cells from 17 cell types (categories). To produce our data set, we sample randomly $2,000$ cells from 10 cell types (200 cells for each type) and select as our features the expression level of the top 500 most highly variable genes across the $2,000$ cells in our sample.

6. *Segmentation*: This is an image segmentation data set, which is provided at UCI machine learning repository[9] at https://archive.ics.uci.edu/ml/datasets/Image+Segmentation. Each sample represents an image described by 19 high-level and man-crafted numeric-valued attributes.

Note S2: Comparison with Seurat clustering

Single-cell clustering is indeed an area where some of the gold standard methods are based on applying community detection to graphs derived from cell features (e.g., gene expression levels) by using the Louvain algorithm to maximize modularity. Seurat[10] is such a graph-based clustering approach, where a kNN graph is constructed from the PCA decomposition of the original feature vectors, and the obtained graph is then partitioned into communities (corresponding to cell types) by Louvain modularity maximization.

It is important to remark that there is a fundamental distinction between the Seurat setting and our work. While our method (CkNN+GCN) addresses a classification problem (supervised setting, in which some class labels are known as ground truths and used in the training), Seurat solves a clustering problem (unsupervised setting, in which there are no class labels available on which to train). Clustering aims to group similar samples together and dissimilar samples into distinct groups based on the similarity between their features[11]. Seurat clustering involves three steps: (i) compute the principal components of the feature vectors, and select the top T principal components based on a choice of p, the ratio of explained variance to total variance; (ii) construct a kNN graph based on the Euclidean distance between the vectors defined by the top T principal components of each sample; and (iii) perform community detection on the kNN graph using Louvain modularity maximization. In this process, several hyperparameters are chosen, including the ratio p, which determines the number of principal components in step (i), and the number of neighbors k in the kNN graph in step (ii). The final result of Seurat is a partition of the data set into clusters ('graph communities') derived intrinsically from properties of the data.

Our method (CkNN+GCN), on the other hand, attempts a classification task where we leverage both the features and a feature-derived CkNN graph of appropriate edge density to train the weights of a GCN in order to maximize its classification power. Our use of GCN and CkNN is distinctive in this setting, as is the optimization of the edge density of the graph to maximize the quality of the classification. Given that the objectives of Seurat and our method are different, it is not straightforward to compare both approaches, but we have produced a setting to compare both methods. In particular, we have devised a comparison between our CkNN+GCN

method and two Louvain-based clustering methods: Seurat (PCA+kNN+Louvain) and a simpler application of Louvain to a kNN graph of features (kNN+Louvain) without applying PCA in the first step. These three methods are compared to a simple kNN classifier (kNNC).

To compare the methods, we use the labels in the training and validation sets (defined as above in our CkNN+GCN experiments) as ground truths, and tune the hyperparameters k and p (k is grid-searched over $[2,4,8,16,32,64]$ and p is grid-searched over $[0.5,0.6,0.7,0.8,0.9]$) to maximize the similarity between the obtained clusters and the ground truth partitions. Once the hyperparameters have been optimized, we then use each method to cluster the data and we compute the quality of the clustering against the test set. To evaluate the quality of the clustering we use two standard measures: the Adjusted Rand Index (ARI) and the Normalized Mutual Information (NMI). Both of these measures are normalized between 0 (random assignment) and 1 (perfect agreement), with higher values signifying better assignment. Our results are presented in Table S6. Our results show that our method (CkNN+GCN) performs better on average than both Louvain-based clustering methods on our data sets. Yet CkNN+GCN does not always outperform the other methods; in particular, Seurat is the best on the Cell data set. This might reflect particularities of the Cell data set, which contains high-dimensional vectors with high levels of noise that might benefit from the effective dimensionality reduction and filtering that PCA enforces. On the other hand, CkNN+GCN has been kept as a broad-purpose method, i.e., not optimized for a particular type of data. For instance, we use default values for some GCN hyperparameters (learning rate, number of hidden units, drop out rate) without optimizing them on each data set. The aim is to provide robust outcomes across diverse data sets, as shown in Table S6. Hence, there is room to potentially optimize our method (CkNN+GCN) specifically for single-cell genomics, but we feel this falls beyond the scope of our current work, and will be investigated in future research.

Still, we would like to remark that clustering and classification algorithms are not directly comparable since they have different objectives and learning contexts. Nonetheless, we hope that our additional experiments provide some insight into the comparison.

Note S3: Code availability

We provide the data sets and code for geometric graph construction at https://github.com/haczqyf/ggc. The code for Graph Convolutional Networks (GCNs) is provided by the authors of[12] at https://github.com/tkipf/gcn. The code for kNN Classification (kNNC), Support Vector Machine (SVM) and Random Forest (RF) can be found at https://scikit-learn.org/stable/ from scikit-learn[5]. The code for Spielman-Srivastava sparsification algorithm (SSSA) is available at https://epfl-lts2.github.io/gspbox-html/doc/utils/gsp_graph_sparsify.html from Graph Signal Processing Toolbox[13].

Note S4: Algorithm complexity

For a graph $\mathcal{G} = (\mathcal{V}, \mathcal{E})$ with N nodes $v_i \in \mathcal{V}$ and $|\mathcal{E}|$ edges $(v_i, v_j) \in \mathcal{E}$, the time complexity for GCN, i.e., to evaluate Equation (9), is $O(|\mathcal{E}|FHC)$[12], where $|\mathcal{E}|$ is the number of graph edges, F is the dimension of the feature space, H is the number of units in the hidden layer and C is the number of classes in the ground truth. Hence the computational complexity for GCN is linear in the number of graph edges. For the geometric graph construction, a brute force approach to compute exactly a geometric graph (i.e., the kNN-type graphs) has time complexity $O(FN^2)$. However, fast approximate kNN graph algorithms were proposed to reduce this time complexity. We mention two examples: (i) Ref.[14] proposes an algorithm with complexity $O(FN^t)$ with $1 < t < 2$, and (ii) Ref.[15] proposes an algorithm that uses locality sensitive hashing, which has complexity $O\left(FN^{1/c^2+o(1)}\right)$ where $c = 1 + \epsilon > 1$. For a thorough list of approximate kNN algorithms, see https://github.com/stephenleo/adventures-with-ann/. Regarding spectral sparsification, the algorithm is nearly linear with time complexity $\tilde{O}(|\mathcal{E}|)$[16], where the $\tilde{O}$ notation ignores logarithmic factors. Finally, for the MST construction, we use the Kruskal algorithm implemented in Scipy with time complexity $O(|\mathcal{E}|\log N)$.

Note S5: Run time and memory requirements

To give a sense of run times and memory requirements for our algorithm, we summarize briefly the numbers for the Cora data set, which presents the worst-case run times and storage requirements among our seven examples. Indeed, we find that Cora has the longest run times, consistent with the algorithmic complexity in Note S4, since Cora has the largest number of nodes and highest dimensions. For graph construction, creating and storing in disk all the graphs during the optimization of the hyperparameter takes around 13 hours with a maximum used memory around 3G. However, our algorithm can be further optimized since the graphs do not have to be stored and could be created and used on the fly to save memory usage and access time.

Furthermore, over-dense graphs could be avoided altogether since the optimized graphs usually are relatively sparse. Indeed, we find that the graphs with optimal accuracy have densities on the order of $0.005 - 0.05$ of the total number of possible edges (see Table S3), and for densities above ~ 0.1 the accuracy drops below the accuracy of an MLP. For higher densities, the accuracy consistently degrades towards the random assignment limit. Therefore the grid search of the hyperparameter can be restricted to low density graphs, and dense graphs do not have to be stored or computed. The search for the optimal hyperparameter can be further aided with a bisection scheme and could be parallelized to improve the efficiency of the optimization.

For a thorough description of the complexity of the different blocks of our algorithm (GCN, kNN and MST graph constructions, and spectral sparsification) see Note S4. For each value of the hyperparameter, we run a GCN 10 times from 10 random initializations. The cost of each GCN is moderate: the complexity of GCN scales nearly linearly with the number of edges of the graph. The cost of constructing kNN-type graphs (originally of $O(N^2)$) can also be reduced to nearly linear in the number of nodes with approximation algorithms. Sparsification is also nearly linear, as shown by Spielman. Hence the methodology has the potential to be applied to relatively large graphs with further code optimization. For instance, each GCN for Cora takes typically less than 7 minutes for relatively sparse graphs ($k \leq 200$), and each graph sparsification takes less than 2 minutes.

Comparing to the Louvain-based methods, there is the same complexity for the kNN graph construction, whereas the run time complexity of Louvain optimization is $O(N \log^2 N)$. For Seurat, there is the additional cost of performing PCA to extract the top T principal components, with complexity $O(N^2 T)$ (inherited from randomized SVD). Thus, the run time complexity and memory requirements of the Louvain-based methods are comparable to those of our method.

Supplemental Tables and Figures

Table S1: Summary statistics of the data sets in our study.

Data sets	Type	Samples (N)	Features (F)	Classes (C)	Train/Validation/Test
Constructive	Stochastic block model	$1,000$	500	10	$50/100/850$
Cora	Text (Bag-of-words)	$2,485$	$1,433$	7	$119/253/2,113$
AMiner	Text (Bag-of-words)	$2,072$	500	7	$98/212/1,762$
Digits	Images (Grayscale pixels)	$1,797$	64	10	$80/189/1,528$
FMA (songs)	Music track features	$2,000$	140	8	$96/204/1,700$
Brain cell types	Single-cell transcriptomics	$2,000$	500	10	$100/200/1,700$
Segmentation	Image features	$2,310$	19	7	$112/234/1,964$

Table S2: Classification accuracy (in percent) on the test set (average and standard deviation over 10 runs with random initializations) for 7 data sets with 8 classifiers (four graph-less methods; GCN with four graph constructions).

Classifier	Constructive	Cora	AMiner	Digits	FMA	Cell	Segmentation
MLP = GCN (No graph)	42.1 ± 1.2	54.2 ± 1.7	54.4 ± 1.1	82.0 ± 1.3	34.3 ± 0.8	79.5 ± 3.0	72.0 ± 2.4
kNNC	31.4 ± 0.0	38.2 ± 0.0	28.0 ± 0.0	88.3 ± 0.0	30.6 ± 0.0	58.7 ± 0.0	68.8 ± 0.0
SVM	40.0 ± 0.0	55.9 ± 0.0	51.4 ± 0.0	87.7 ± 0.0	35.3 ± 0.0	81.5 ± 0.0	87.7 ± 0.0
RF	36.3 ± 1.0	56.1 ± 1.2	47.7 ± 1.5	83.0 ± 0.5	33.0 ± 0.9	88.0 ± 0.7	88.8 ± 0.7
GCN (kNN)	53.9 ± 0.9	66.4 ± 0.6	59.2 ± 1.3	92.0 ± 0.4	35.6 ± 1.0	83.8 ± 1.6	83.5 ± 0.7
GCN (MkNN)	45.2 ± 1.6	64.1 ± 0.4	61.8 ± 0.8	93.2 ± 0.3	35.6 ± 0.7	84.0 ± 2.0	83.0 ± 0.6
GCN (CkNN)	51.1 ± 1.3	66.6 ± 0.4	61.6 ± 0.8	93.4 ± 0.3	36.0 ± 0.8	84.0 ± 2.1	83.9 ± 0.6
GCN (RMST)	45.9 ± 1.5	64.8 ± 0.6	61.5 ± 1.3	89.3 ± 0.5	35.4 ± 0.7	84.9 ± 1.1	83.0 ± 1.6

Table S3: Selected density parameters and density of constructed graphs in the graph densification process (Figure S1).

Data set	kNN		MkNN		CkNN ($\delta = 1$)		RMST ($k = 1$)	
	k^*	Density	k^*	Density	k^*	Density	γ^*	Density
Constructive	9	0.01741	104	0.02101	33	0.00920	0.07421	0.02724
Cora	12	0.00842	39	0.00436	74	0.01476	0.02924	0.01242
AMiner	8	0.00748	199	0.01786	199	0.03852	0.02317	0.00859
Digits	5	0.00404	39	0.01400	33	0.01564	0.00346	0.00117
FMA	1	0.00100	2	0.00103	13	0.00398	0.00146	0.00107
Cell	1	0.00100	8	0.00133	41	0.00753	0.00320	0.00124
Segmentation	7	0.00387	20	0.00637	12	0.00447	0.03423	0.00117

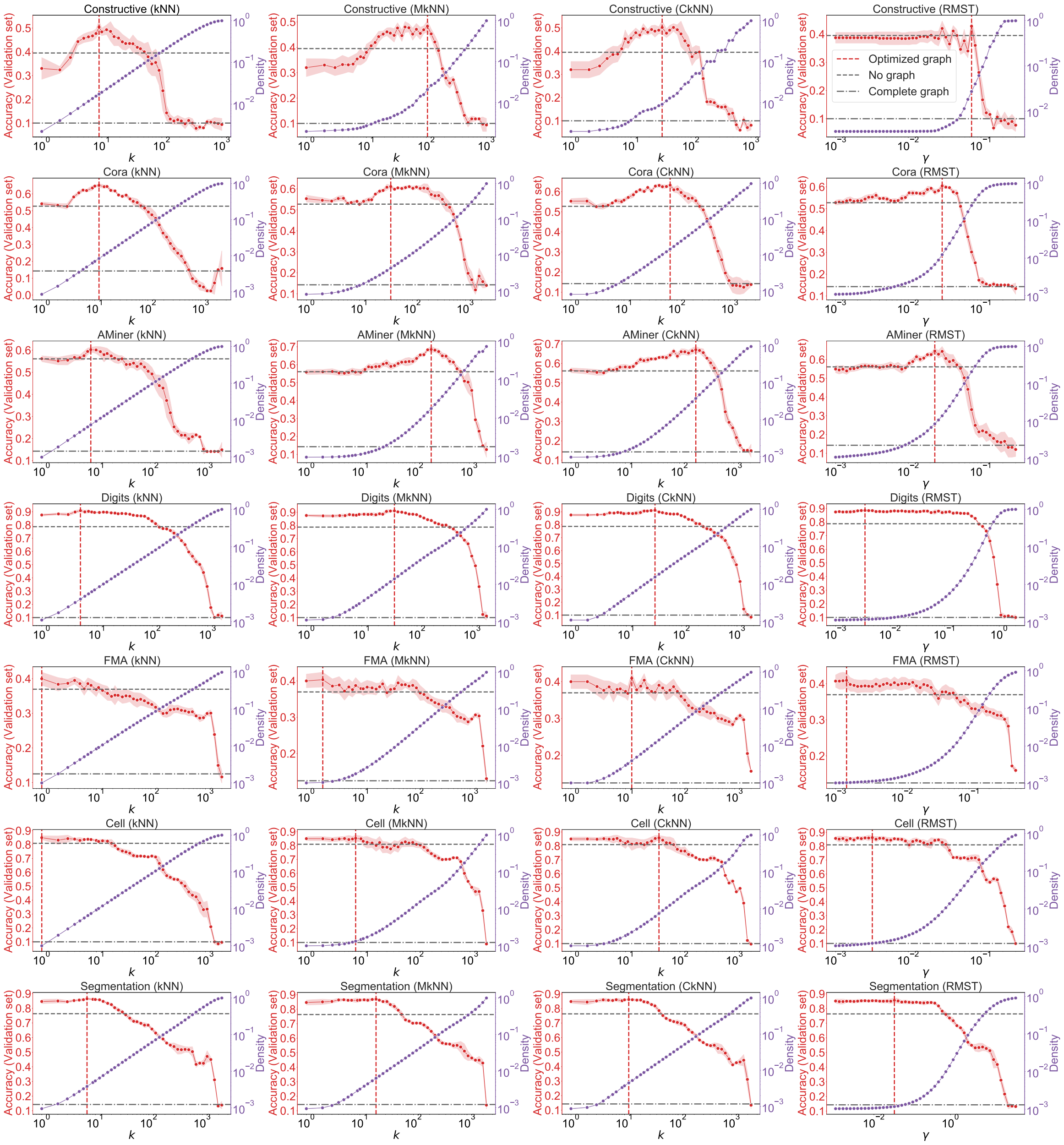

Figure S1: Graph construction search in the densification process. The red line indicates the mean classification accuracy on the validation set of 10 runs with random weight initializations as a function of the density parameter. The red shaded regions denote the standard deviation. The mean classification accuracy on the validation of two limiting cases (no graph and complete graph) are added as well. The red vertical line indicates the optimized graph. The purple line shows the densities of the constructed graphs.

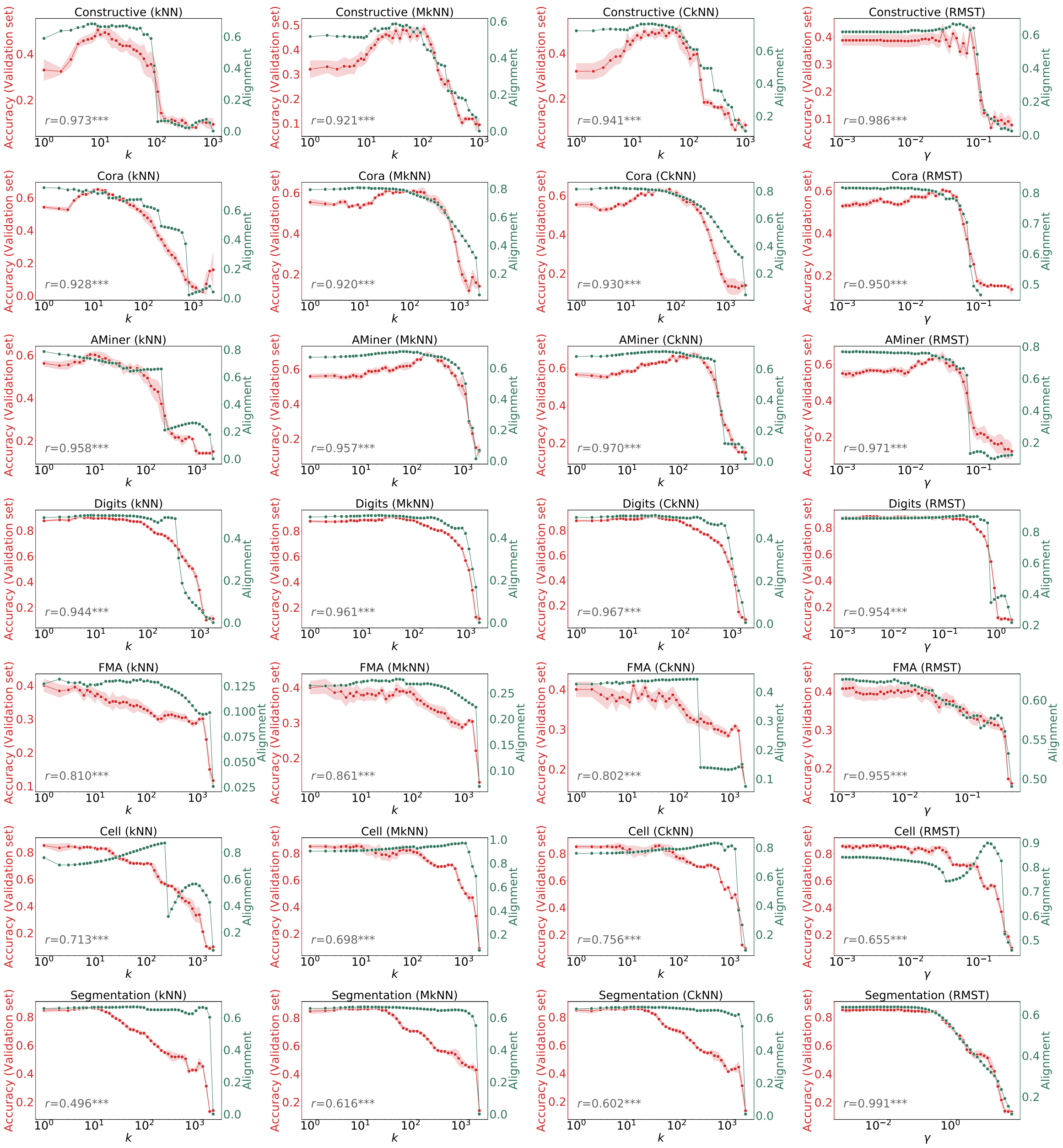

Figure S2: The red line indicates the mean classification accuracy on the validation set of 10 runs with random weight initializations as a function of the density parameter. The red shaded regions denote the standard deviation. The green line indicates the alignment. We report the Pearson correlation coefficients and p-values between mean accuracy and alignment. *p-value < 0.05,** p-value < 0.01,*** p-value < 0.001.

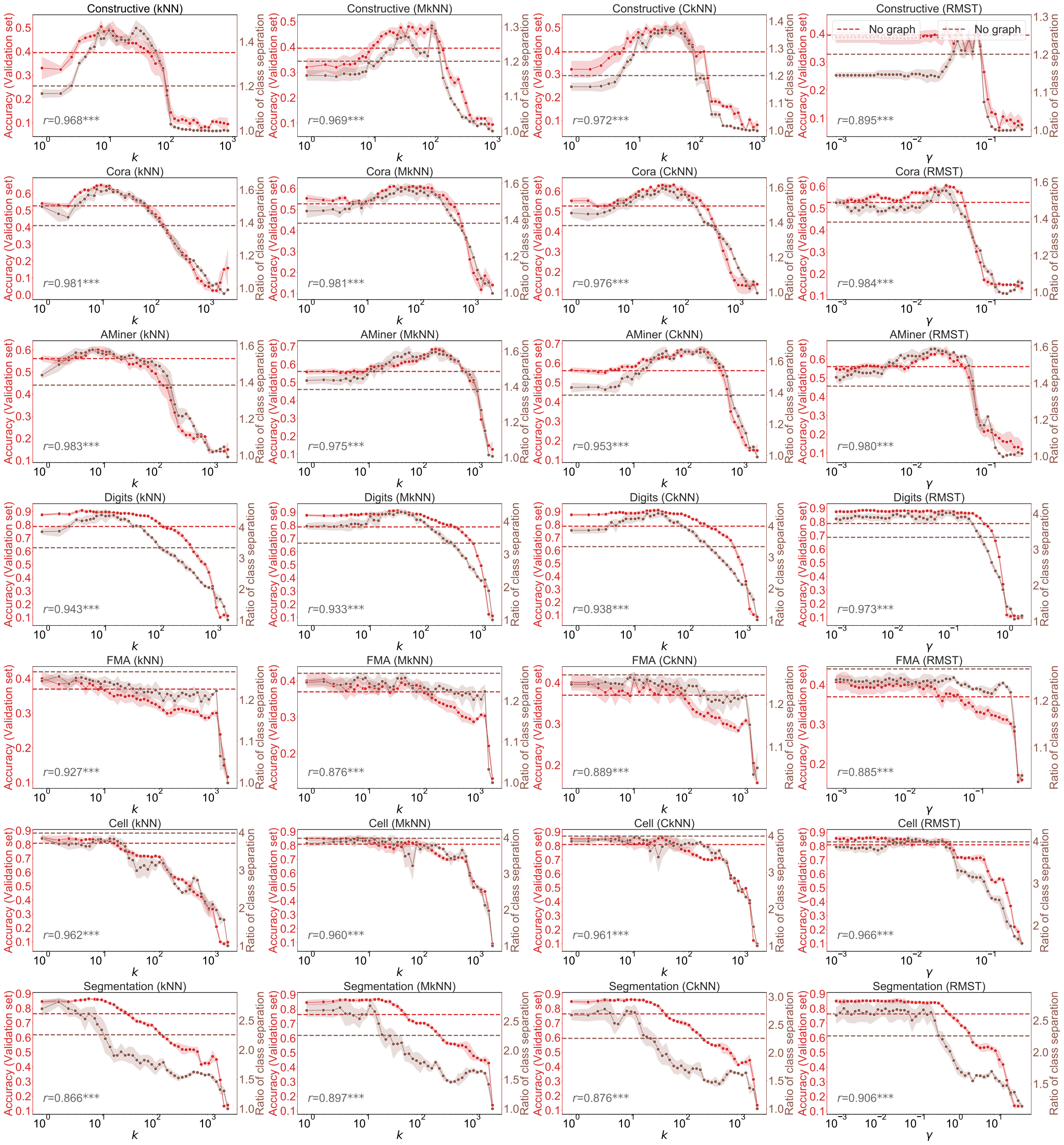

Figure S3: The red line indicates the mean classification accuracy on the validation set of 10 runs with random weight initializations as a function of the density parameter. The red shaded regions denote the standard deviation. The red dashed line represents the mean classification accuracy on the validation of no graph case. The brown line shows the ratio of class separation in the densification process. The brown shaded regions denote the standard deviation. The brown dashed line represents the ratio of class separation of no graph case. We report the Pearson correlation coefficients and p-values between mean accuracy and mean ratio of class separation. *p-value < 0.05,** p-value < 0.01,*** p-value < 0.001.

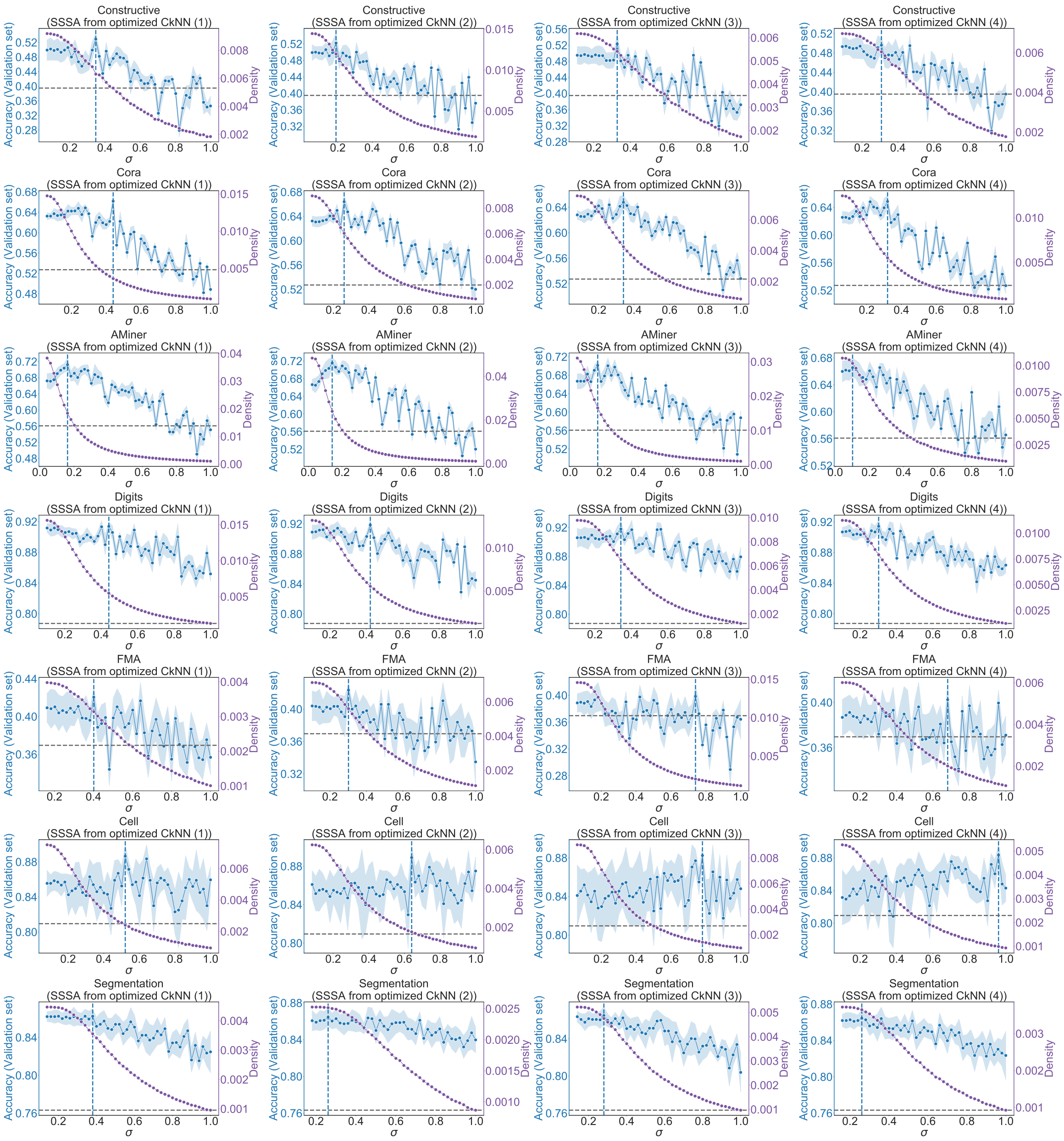

Figure S4: Graph construction search in the sparsification process. The blue line indicates the mean classification accuracy on the validation set of 10 runs with random weight initializations as a function of the density parameter. The blue shaded regions denote the standard deviation. The mean classification accuracy on the validation of no graph is added as well. The blue vertical line indicates the optimized graph on the validation set. The purple line shows the densities of the sparsified graphs.

Table S4: Comparison between optimized CkNN and sparsification of optimized CkNN graphs (Figure S4).

**Top 4 CkNN graphs on validation set**	**Data set**	**Optimized CkNN**				**Sparsification of optimized CkNN**			
		k^*	Edge density	$\langle$Degree$\rangle$	Accuracy (Test)	σ^*	Edge density	$\langle$Degree$\rangle$	Accuracy (Test)
**(1)**	Constructive	33	0.00920	9.2	51.1	0.3479	0.00630	6.3	51.6
	Cora	74	0.01476	36.7	66.6	0	0.01476	36.7	66.6
	AMiner	199	0.03852	79.8	61.6	0.1618	0.01840	38.1	62.5
	Digits	33	0.01564	28.1	93.4	0	0.01564	28.1	93.4
	FMA	13	0.00398	8.0	36.0	0	0.00398	8.0	36.0
	Cell	41	0.00753	15.0	84.0	0.5212	0.00240	4.8	85.0
	Segmentation	12	0.00447	10.3	83.9	0.3806	0.00356	8.2	84.0
	**Average improvement**				(+8.3)				(+8.7)
**(2)**	Constructive	51	0.01445	14.4	51.8	0.1898	0.01197	12.0	53.6
	Cora	46	0.00897	22.3	66.3	0	0.00897	22.3	66.3
	AMiner	233	0.04838	100.2	61.3	0.1418	0.02396	49.6	63.6
	Digits	28	0.01319	23.7	93.2	0.4222	0.00556	10.0	93.2
	FMA	22	0.00713	14.3	35.2	0.3018	0.00561	11.2	35.8
	Cell	35	0.00625	12.5	83.6	0.6409	0.00176	3.5	86.9
	Segmentation	7	0.00253	5.8	84.0	0.2607	0.00252	5.8	84.2
	**Average improvement**				(+8.1)				(+9.3)
**(3)**	Constructive	16	0.00618	6.2	49.0	0	0.00618	6.2	49.0
	Cora	39	0.00756	18.8	66.8	0	0.00756	18.8	66.8
	AMiner	171	0.03115	64.5	62.1	0.1618	0.01656	34.3	63.5
	Digits	21	0.00978	17.6	92.9	0.3425	0.00650	11.7	93.0
	FMA	41	0.01457	29.1	35.9	0	0.01457	29.1	35.9
	Cell	48	0.00904	18.1	81.9	0.7806	0.00141	2.8	84.1
	Segmentation	14	0.00522	12.1	83.8	0	0.00522	12.1	83.8
	**Average improvement**				(+7.7)				(+8.2)
**(4)**	Constructive	22	0.00697	7.0	51.2	0.3084	0.00605	6.0	51.4
	Cora	63	0.01246	30.9	65.9	0	0.01246	30.9	65.9
	AMiner	78	0.01071	22.2	62.0	0.1019	0.01021	21.1	62.1
	Digits	24	0.01125	20.2	92.9	0	0.01125	20.2	92.9
	FMA	19	0.00601	12.0	34.5	0.6808	0.00201	4.0	35.2
	Cell	30	0.00527	10.5	81.8	0.9601	0.00099	2.0	85.3
	Segmentation	10	0.00372	8.6	83.9	0.2607	0.00365	8.4	84.1
	**Average improvement**				(+7.7)				(+8.3)

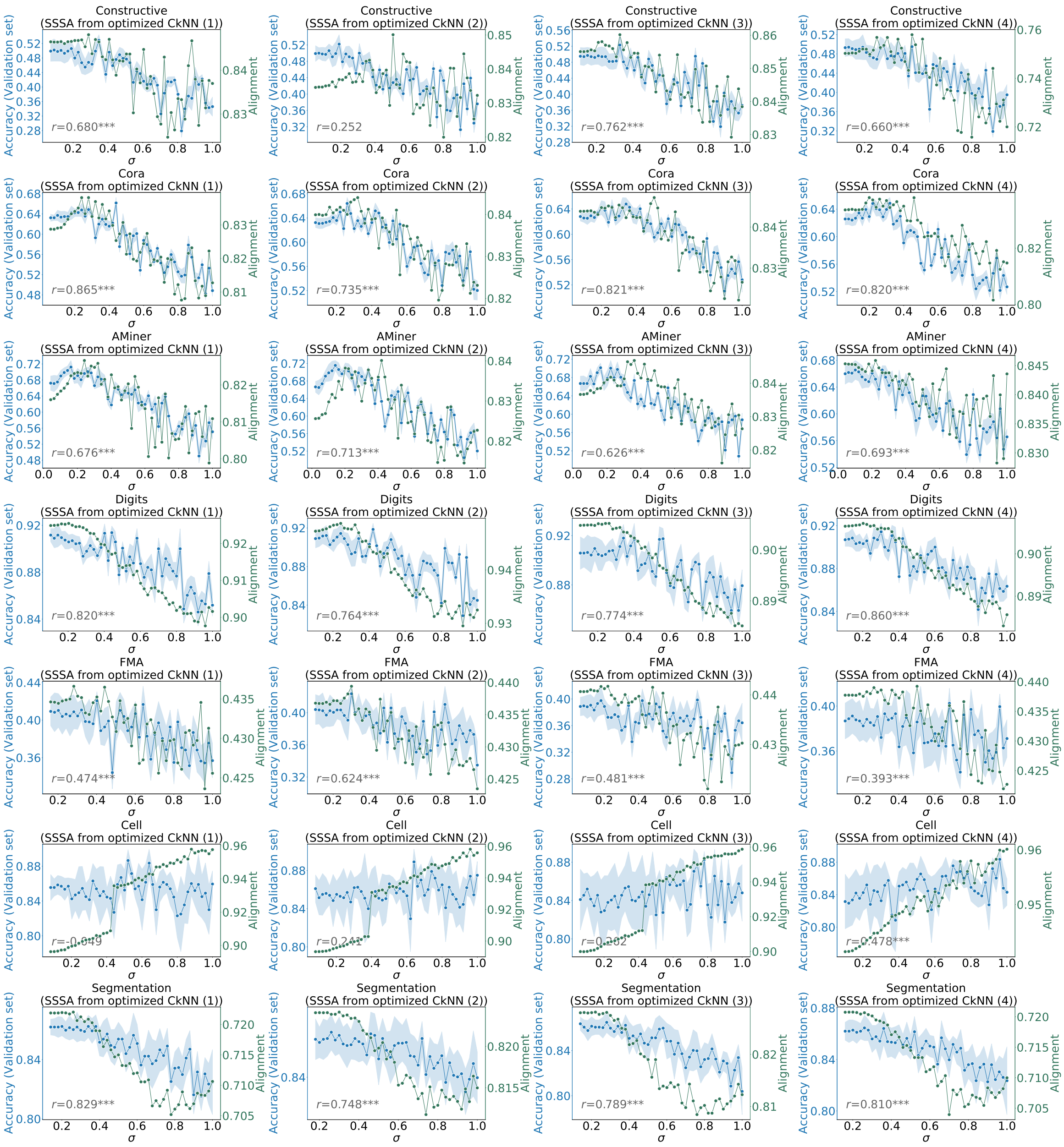

Figure S5: The blue line indicates the mean classification accuracy on the validation set of 10 runs with random weight initializations as a function of the density parameter. The blue shaded regions denote the standard deviation. The green line indicates the alignment. We report the Pearson correlation coefficients and p-values between mean accuracy and alignment. *p-value < 0.05,** p-value < 0.01,*** p-value < 0.001.

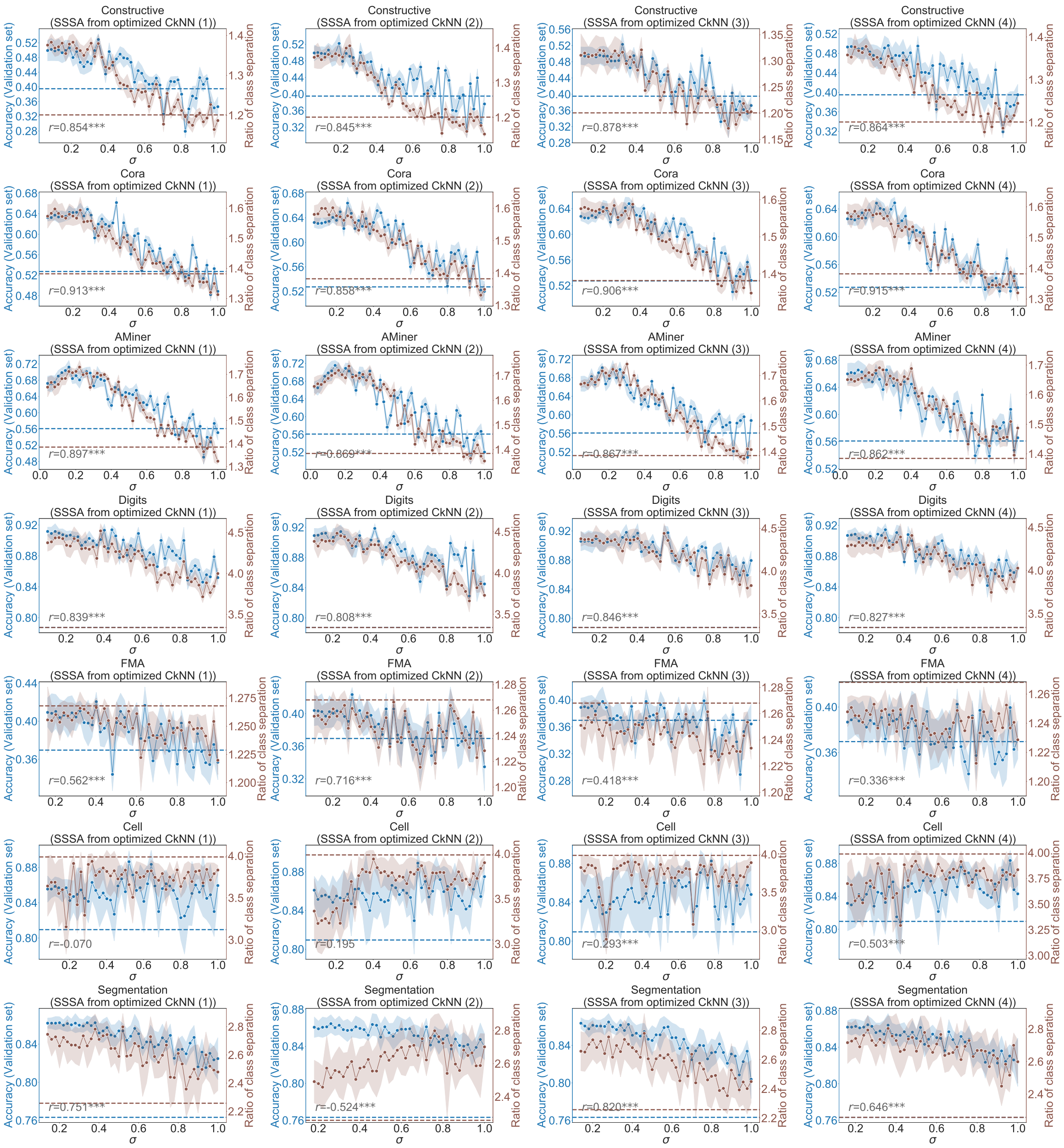

Figure S6: The blue line indicates the mean classification accuracy on the validation set of 10 runs with random weight initializations as a function of the density parameter. The blue shaded regions denote the standard deviation. The blue dashed line represents the mean classification accuracy on the validation of no graph case. The brown line shows the ratio of class separation in the sparsification process. The brown shaded regions denote the standard deviation. The brown dashed line represents the ratio of class separation of no graph case. We report the Pearson correlation coefficients and p-values between mean accuracy and mean ratio of class separation. *p-value < 0.05,** p-value < 0.01,*** p-value < 0.001.

Table S5: Classification accuracy (test set) obtained with three free feature-only methods: MLP, kNN+LDS+GCN[6], and CkNN+GCN (this paper). For information, we also include the accuracy achieved by GCN applied to features together with the additional graph given in the original data set (when available).

Method	Cora	AMiner	Digits	FMA	Cell	Segmentation	Avg. improvement
MLP	54.2	54.4	82.0	34.3	79.5	72.0	—
kNN+LDS+GCN[6]	**69.0**	59.3	**94.6**	**36.2**	80.0	**83.9**	(+7.8)
CkNN + GCN (this paper)	66.6	**61.6**	93.4	36.0	**84.0**	**83.9**	**(+8.2)**
Additional original graph + GCN	*81.1*	*74.8*	–	–	–	–	–

Table S6: Quality of assignments (test set) obtained by a simple kNN classifier (kNNC), two Louvain-based methods (kNN+Louvain, Seurat), and our method (CkNN+GCN). The hyperparameters of all methods (kNNC, Louvain methods, and CkNN+GCN) were optimized on the training and validation sets. Two quality measures are computed (ARI and NMI), both normalized between 0 and 1, with higher values indicating better agreement with the ground truth of the test set. The average improvement with respect to the kNNC is also presented in the last column.

ARI							
**Method**	**Cora**	**AMiner**	**Digits**	**FMA**	**Cell**	**Segmentation**	**Avg. improvement**
kNNC	0.090	0.036	0.766	0.087	0.434	0.456	—
kNN+Louvain	0.337	0.301	0.840	0.086	0.721	0.273	0.115
Seurat=PCA+kNN+Louvain	0.321	0.305	**0.888**	0.086	**0.822**	0.189	0.124
CkNN+GCN (this paper)	**0.382**	**0.348**	0.863	**0.108**	0.767	**0.702**	**0.217**
**NMI**							
**Method**	**Cora**	**AMiner**	**Digits**	**FMA**	**Cell**	**Segmentation**	**Avg. improvement**
kNNC	0.130	0.131	0.806	0.123	0.644	0.532	—
kNN+Louvain	0.386	0.323	0.892	0.125	0.811	0.513	0.114
Seurat=PCA+kNN+Louvain	0.391	0.356	**0.904**	0.118	**0.904**	0.350	0.110
CkNN+GCN (this paper)	**0.408**	**0.409**	0.889	**0.147**	0.854	**0.753**	**0.183**

Supplemental References

[1] Qian, Y., Expert, P., Rieu, T., Panzarasa, P., and Barahona, M. (2021). Quantifying the alignment of graph and features in deep learning. *IEEE Transactions on Neural Networks and Learning Systems*, https://doi.org/10.1109/TNNLS.2020.3043196.

[2] Sen, P., Namata, G., Bilgic, M., Getoor, L., Galligher, B., and Eliassi-Rad, T. (2008). Collective classification in network data. *AI Magazine*, 29(3):93–93, https://doi.org/10.1609/AIMAG.V29I3.2157.

[3] Qian, Y., Rong, W., Jiang, N., Tang, J., and Xiong, Z. (2017). Citation regression analysis of computer science publications in different ranking categories and subfields. *Scientometrics*, 110(3):1351–1374, https://doi.org/10.1007/S11192-016-2235-4.

[4] Tang, J., Zhang, J., Yao, L., Li, J., Zhang, L., and Su, Z. (2008). Arnetminer: extraction and mining of academic social networks. In *ACM SIGKDD International Conference on Knowledge Discovery and Data Mining*, https://doi.org/10.1145/1401890.1402008.

[5] Pedregosa, F., Varoquaux, G., Gramfort, A., Michel, V., Thirion, B., Grisel, O., Blondel, M., Prettenhofer, P., Weiss, R., Dubourg, V., et al. (2011). Scikit-learn: Machine learning in python. *Journal of Machine Learning Research*, 12(Oct):2825–2830.

[6] Franceschi, L., Niepert, M., Pontil, M., and He, X. (2019). Learning discrete structures for graph neural networks. In *International Conference on Machine Learning*.

[7] Defferrard, M., Benzi, K., Vandergheynst, P., and Bresson, X. (2017). FMA: A dataset for music analysis. In *International Symposium/Conference on Music Information Retrieval*.

[8] Velmeshev, D., Schirmer, L., Jung, D., Haeussler, M., Perez, Y., Mayer, S., Bhaduri, A., Goyal, N., Rowitch, D. H., and Kriegstein, A. R. (2019). Single-cell genomics identifies cell type-specific molecular changes in autism. *Science*, 364(6441):685–689, https://doi.org/10.1126/SCIENCE.AAV8130.

[9] Dua, D. and Graff, C. (2019). UCI machine learning repository. Irvine, CA: University of California, School of Information and Computer Science, http://archive.ics.uci.edu/ml.

[10] Satija, R., Farrell, J.A., Gennert, D., Schier, A.F. and Regev, A. (2015). Spatial reconstruction of single-cell gene expression data. *Nature Biotechnology*, 33(5), pp.495-502, https://doi.org/10.1038/nbt.3192.

[11] Liu, Z. and Barahona, M. (2020). Graph-based data clustering via multiscale community detection. *Applied Network Science*, 5(1):3, https://doi.org/10.1007/S41109-019-0248-7.

[12] Kipf, T. N. and Welling, M. (2017). Semi-supervised classification with graph convolutional networks. In *International Conference on Learning Representations*.

[13] Perraudin, N., Paratte, J., Shuman, D., Martin, L., Kalofolias, V., Vandergheynst, P., and Hammond, D. K. (2014). Gspbox: A toolbox for signal processing on graphs. *arXiv preprint arXiv:1408.5781*.

[14] Chen, J., Fang, H.R. and Saad, Y. (2009). Fast Approximate kNN Graph Construction for High Dimensional Data via Recursive Lanczos Bisection. *Journal of Machine Learning Research*, 10(9):1989–2012.

[15] Andoni, A. and Indyk, P. (2006). Near-optimal hashing algorithms for approximate nearest neighbor in high dimensions. In *IEEE Symposium on Foundations of Computer Science*, https://doi.org/10.1109/FOCS.2006.49.

[16] Spielman, D. A. and Srivastava, N. (2011). Graph sparsification by effective resistances. *SIAM Journal on Computing*, 40(6):1913–1926, https://doi.org/10.1137/080734029.
